# Hybrid-decoupling-based shared-aperture phased array antenna for Ku/Ka-band with low profile, wide-angle scanning and high isolation

**DOI:** 10.1038/s41598-025-23051-6

**Published:** 2025-11-10

**Authors:** Wenxuan Xie, Changlin Li, Yongzhong Zhu, Yunxue Xu, Bao Xiong

**Affiliations:** 1 Department of Information and Communications, Engineering University of the People’s Armed Police, Xi’an, 710086 China; 2 Department of Information and Communications, Special Police College of the People’s Armed Police, Beijing, 102200 China; 3 Xidian University, National Key Laboratory of Antennas and Microwave Technology, Xi’an, 710071 China

**Keywords:** Mathematics and computing, Physics

## Abstract

This paper presents a low-profile Ku/Ka dual-band shared-aperture phased array antenna (SAPAA) that addresses conventional limitations in profile height, port isolation, and scanning angles through a hybrid-decoupling approach combining array decoupling surfaces (ADS) with defected ground structure (DGS), complemented by structural reuse techniques. The Ka-band elements utilize co-designed array decoupling surface layers integrated with DGS to effectively suppress surface current coupling, achieving wide-angle scanning capability of $\pm 45^\circ$ across the 32–36 GHz frequency band. For the dual-polarized Ku-band elements, feed matching is optimized through cross-shaped slots employing DGS structural reuse, demonstrating orthogonal port isolation exceeding 31 dB within the 14–18 GHz operational band while extending the scanning range to $\pm 55^\circ$ in both E- and H-planes. The antenna features an ultra-thin profile of merely 2.35 mm (0.28$\lambda _h$), with measured inter-band isolation reaching 35 dB in the Ku-band and 18 dB in the Ka-band. Experimental verification confirms the design’s high performance and practical utility, offering a compact solution for multi-band integrated communication systems.

## Introduction

With the multiband collaborative evolution of satellite and radar systems, shared-aperture phased array antennas (SAPAAs) have garnered significant research attention ^1–7^. However, these systems face a fundamental trilemma in practical implementations: *First*, low-profile configurations inherently impose trade-offs between bandwidth and scanning stability ^8–12^; *Second*, spectral co-location constraints typically limit inter-band isolation to less than 30 dB ^13–17^; *Third*, wide-angle scanning requires maintaining structural periodicity under strong mutual coupling effects ^18–24^. Addressing these challenges demands synergistic development of innovative radiator architectures and advanced decoupling mechanisms.

Recent advances in dual-band SAPAA designs have demonstrated various approaches. The waveguide-based self-filtering structure in ^25^ achieved inter-band isolation of 28 dB (Ku-band) and 70 dB (Ka-band) through structural reuse, though with limited $\pm 40^\circ$ Ka-band scanning capability and bandwidth constraints. An improved design in ^26^ extended scanning coverage to $\pm 60^\circ$ (Ku-band) and $\pm 40^\circ$ (Ka-band), but maintained a relatively high profile of 0.8$\lambda _0$ with moderate isolation (15 dB Ku-band, 18 dB Ka-band). The dual-polarized Ku/single-polarized W-band array in ^27^ achieved $\pm 60^\circ$ and $\pm 45^\circ$ scanning respectively, albeit with reduced W-band aperture efficiency due to sparse array configuration. The end-fire dipole implementation in ^28^ supported $\pm 50^\circ$ scanning for both bands, yet suffered from scan blindness induced by strong coupling between Ka-band elements and vertically polarized Ku-band antennas.

State-of-the-art solutions present further innovations. The sector-optimized approach in ^29^ maintained high isolation across $\pm 50^\circ$ scanning through discrete $3^\circ$-spaced sector optimization, though requiring multiple excitation parameter sets that increase system complexity. The single-layer Phoenix Ring metasurface in ^30^ employed cross-shaped parasitic patches to suppress co-polarized coupling, enabling $\pm 60^\circ$ scanning, but exhibited H-plane gain degradation due to triangular array sparsity. The grid-based dual-patch structure in ^31^ achieved remarkable 0.022$\lambda _l$ profile through embedded millimeter-wave arrays, representing one of the thinnest implementations, though constrained to $\pm 45^\circ$ scanning with compromised gain performance.

This paper proposes a Ku/Ka dual-band SAPAA based on a stacked hybrid-decoupling architecture, which innovatively employs structural reuse technology and co-design of array decoupling surfaces (ADS) with defected ground structure (DGS) to achieve a breakthrough in low-profile configuration, high isolation, and wide-angle scanning. Some of the key contributions of the proposed work are as follows:

- The proposed antenna features a compact volume with 2.35 mm (0.28$\lambda _h$) low profile, achieved through multiple structural reuse techniques that ingeniously arrange dual-polarized Ku-band elements in a 2 $\times$ 2 mirrored configuration with single-polarized Ka-band elements.
- The designed SAPAA demonstrates broadband performance through co-optimization of decoupling surfaces and H-shaped feeding slots, enabling Ka-band operation from 32–36 GHz while covering 14–18 GHz for Ku-band, thus providing wide bandwidth for diverse communication and radar applications.
- Through the hybrid decoupling structure and structural reuse, simultaneous electromagnetic isolation for both in-band and cross-band operations is achieved. Specifically, in the Ku-band, the in-band isolation exceeds 35 dB while the cross-band isolation surpasses 36.3 dB. For the Ka-band, the in-band and cross-band isolations exceed 18 dB and 17.7 dB, respectively. This design effectively preserves signal integrity and significantly suppresses inter-channel interference.
- Through joint simulation optimization of radiating elements and decoupling structures, the design achieves scanning range breakthroughs under low-profile constraints: $\pm 55^\circ$ 2D scanning in Ku-band (gain variation <3.3 dB in E/H planes) and stable $\pm 45^\circ$ scanning in Ka-band (gain variation <1.55 dB in E/H planes).

**Fig. 1:**
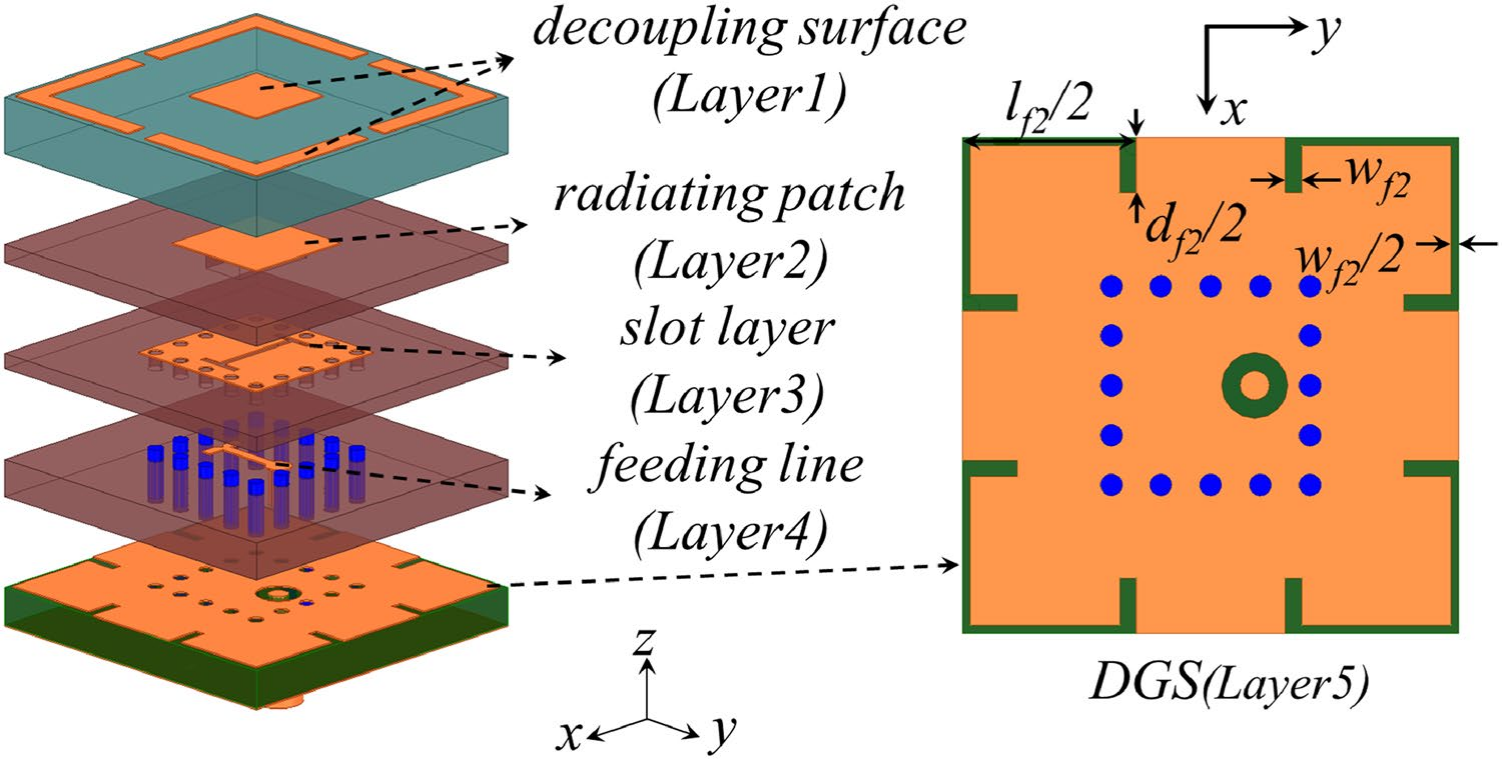
3-D exploded view and DGS view of the Ka-band element.

## Design procedure of the SAPAA

### Design of Ka-band element and decoupling structure

Figure 1 illustrates the preliminary design of the Ka-band element, where a coaxial probe excites the slot on the substrate-integrated waveguide (SIW) cavity, and the energy is radiated through a square patch. The ADS is located at the top layer, formed by a discontinuous square ring surrounding a square patch. By adjusting the patch dimensions and the thickness of the top dielectric substrate, a reflected wave with equal amplitude but opposite phase to the original coupled wave is generated, achieving coupling cancellation. Specifically, the square patch cancels the primary coupled wave, while the surrounding secondary annular patch suppresses cross-polarization coupling. The DGS is positioned at the bottom layer, where eight L-shaped slots are etched at the four corners of the ground plane to create band-stop characteristics, obstructing the propagation path of coupled currents and improving isolation.

**Fig. 2:**
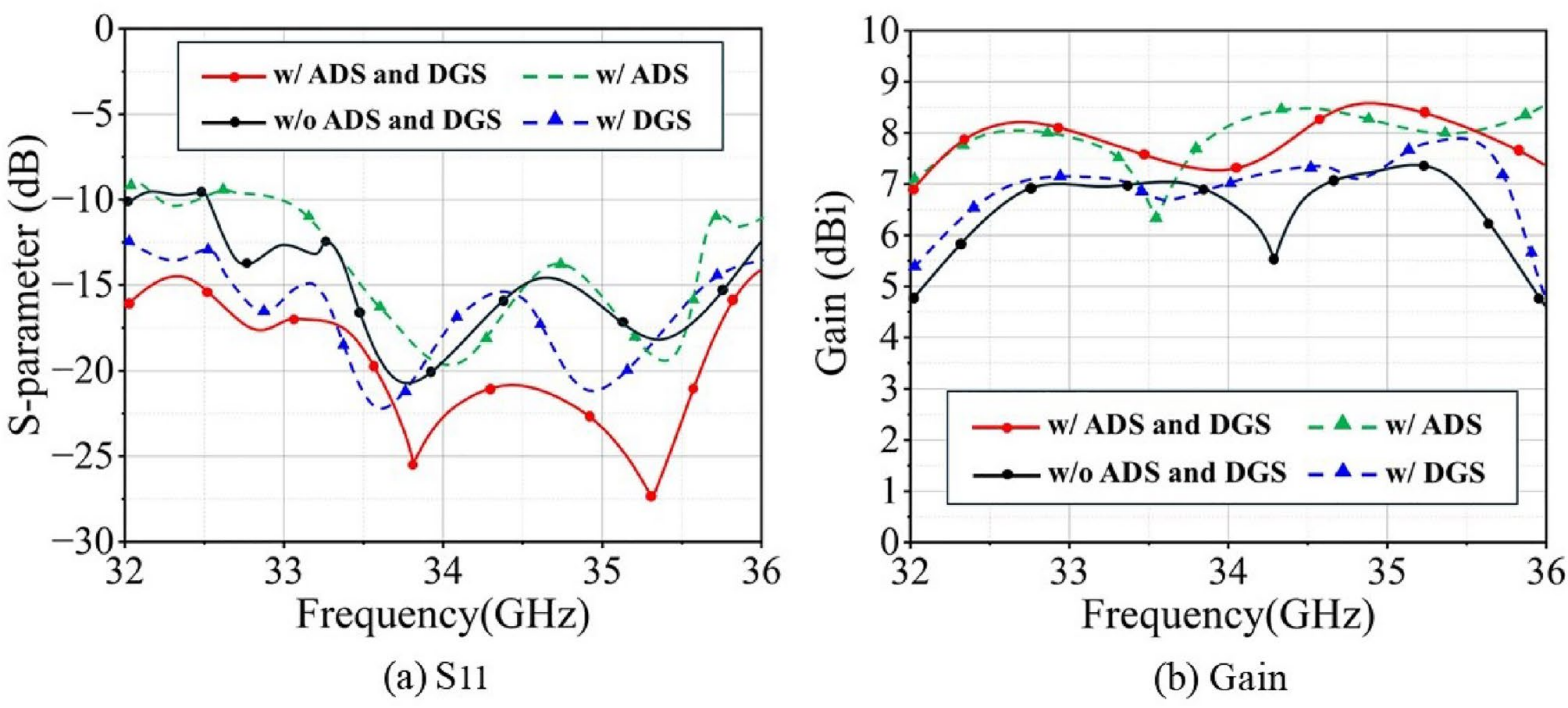
Effects of ADS/DGS decoupling structures on $\text {S}_{11}$ and gain characteristics of Ka-band antenna element.

To evaluate the role of the hybrid decoupling structure at the unit cell level, Fig. 2 presents a comparison of $\text {S}_{11}$ and gain performance for four Ka-band antenna unit configurations: (1) with both ADS and DGS, (2) without any decoupling structure, (3) with ADS only, and (4) with DGS only. To ensure a fair comparison, all results are obtained from simulations of thoroughly tuned and optimized models.

As shown in Fig. 2a, compared to the antenna unit without any decoupling structure, the proposed L-shaped DGS effectively blocks the coupling current paths within the ground plane and suppresses surface wave resonance, thereby significantly expanding the operating bandwidth. In contrast, the standalone ADS structure acts as an additional resonator, whose radiation field constructively superimposes with that of the antenna, contributing to gain enhancement. However, its individual application leads to a reduction in the lower-frequency bandwidth.Through co-optimization of ADS and DGS, the DGS effectively compensates for the low-frequency bandwidth loss caused by ADS, achieving stable impedance matching across the entire band and eliminating the mid-band gain depression. As visible in Fig. 2b, the unit without decoupling exhibits a noticeable gain drop in the 33.8–34.6 GHz range; this depression is effectively eliminated with DGS loading; while ADS further enhances the overall gain level. Their synergistic operation ultimately achieves a peak gain of 8.6 dBi.

**Fig. 3:**
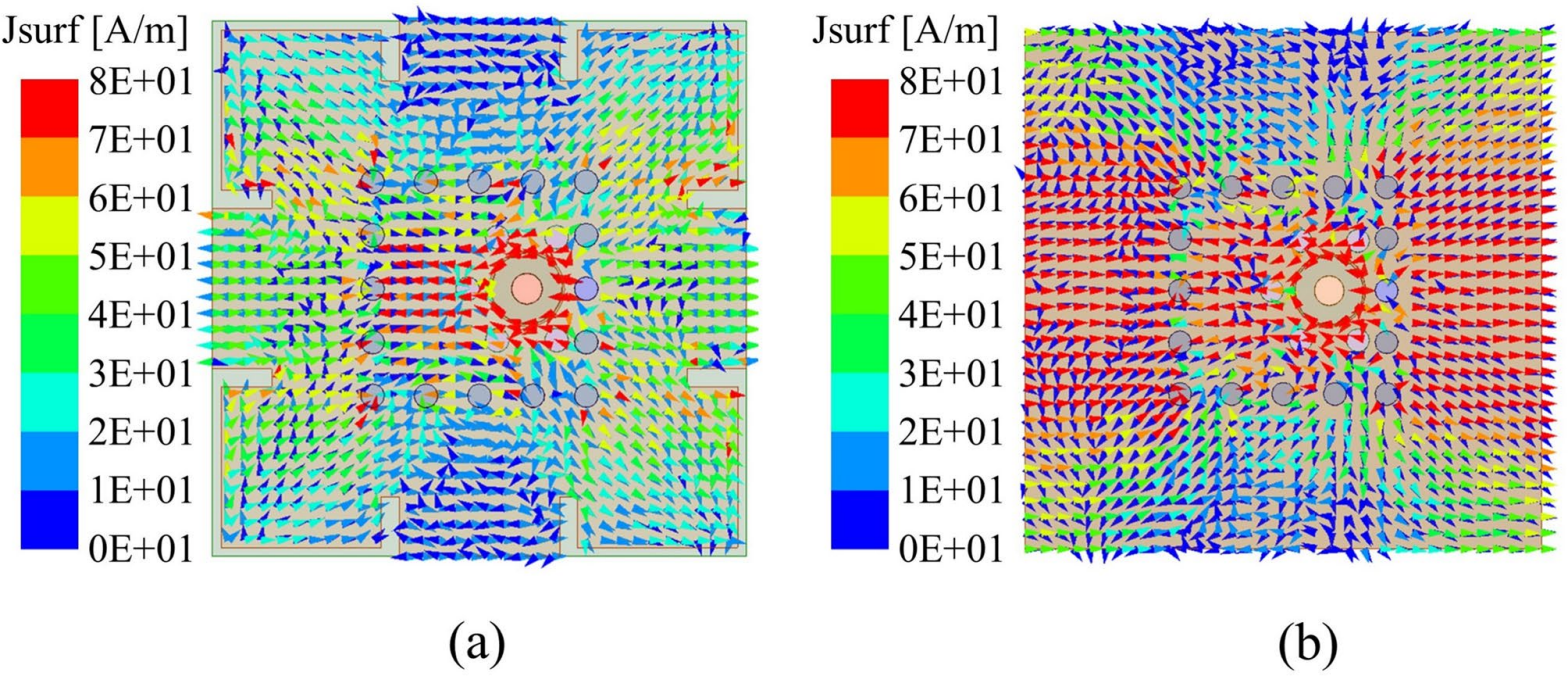
Current distributions simulation results of Ka-band elements (**a**) with and (**b**) without hybrid-decoupling structure.

**Fig. 4:**
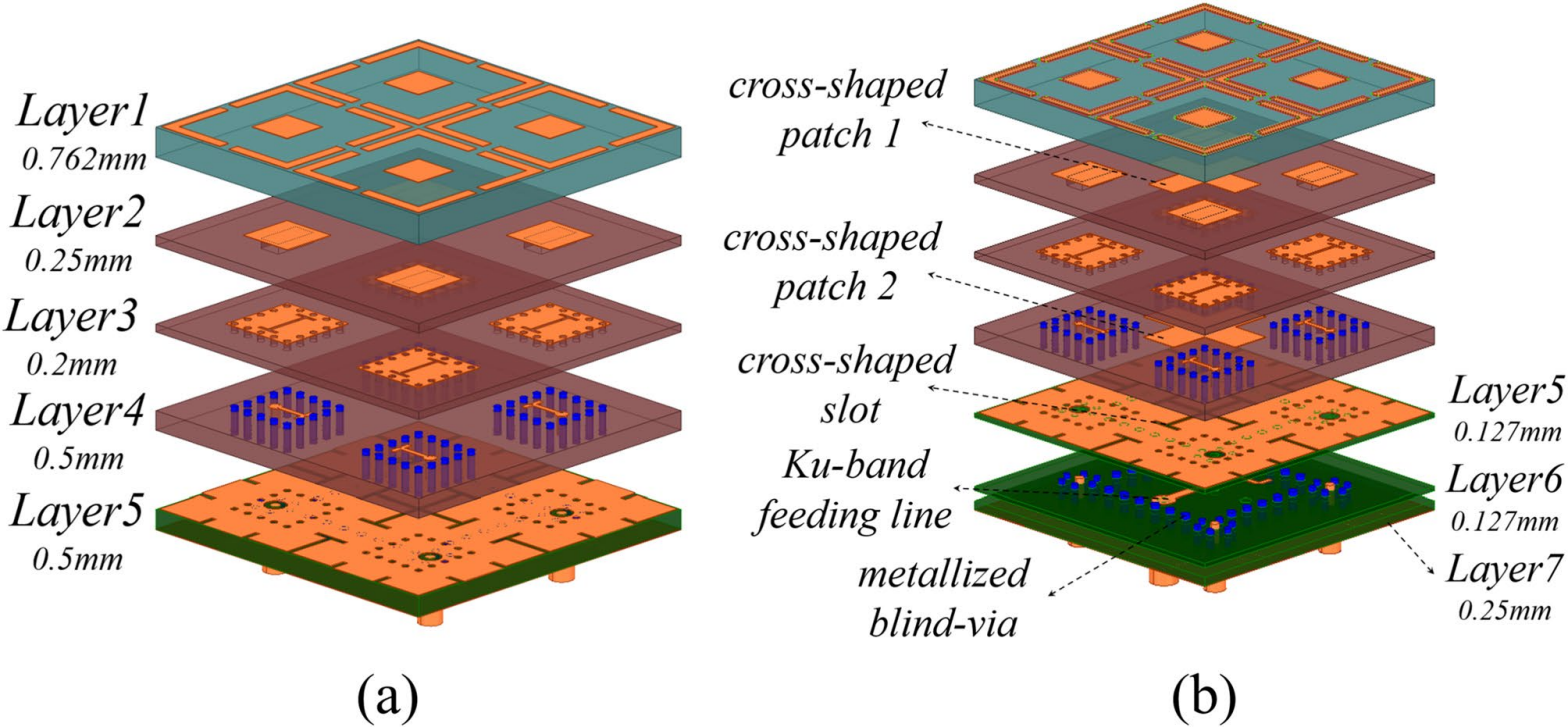
(**a**) The 2 $\times$ 2 Ka-band array and (**b**) the modified Ku-band element.

The current distributions in Fig. 3a,b demonstrate that the hybrid decoupling structure significantly reduces edge currents on the ground plane and effectively suppresses surface wave resonance. This observation further confirms the structure’s substantial contribution to bandwidth extension and gain stabilization at the unit cell level. The decoupling performance of this hybrid structure in antenna arrays and co-aperture designs will be discussed in detail in subsequent sections.

**Fig. 5:**
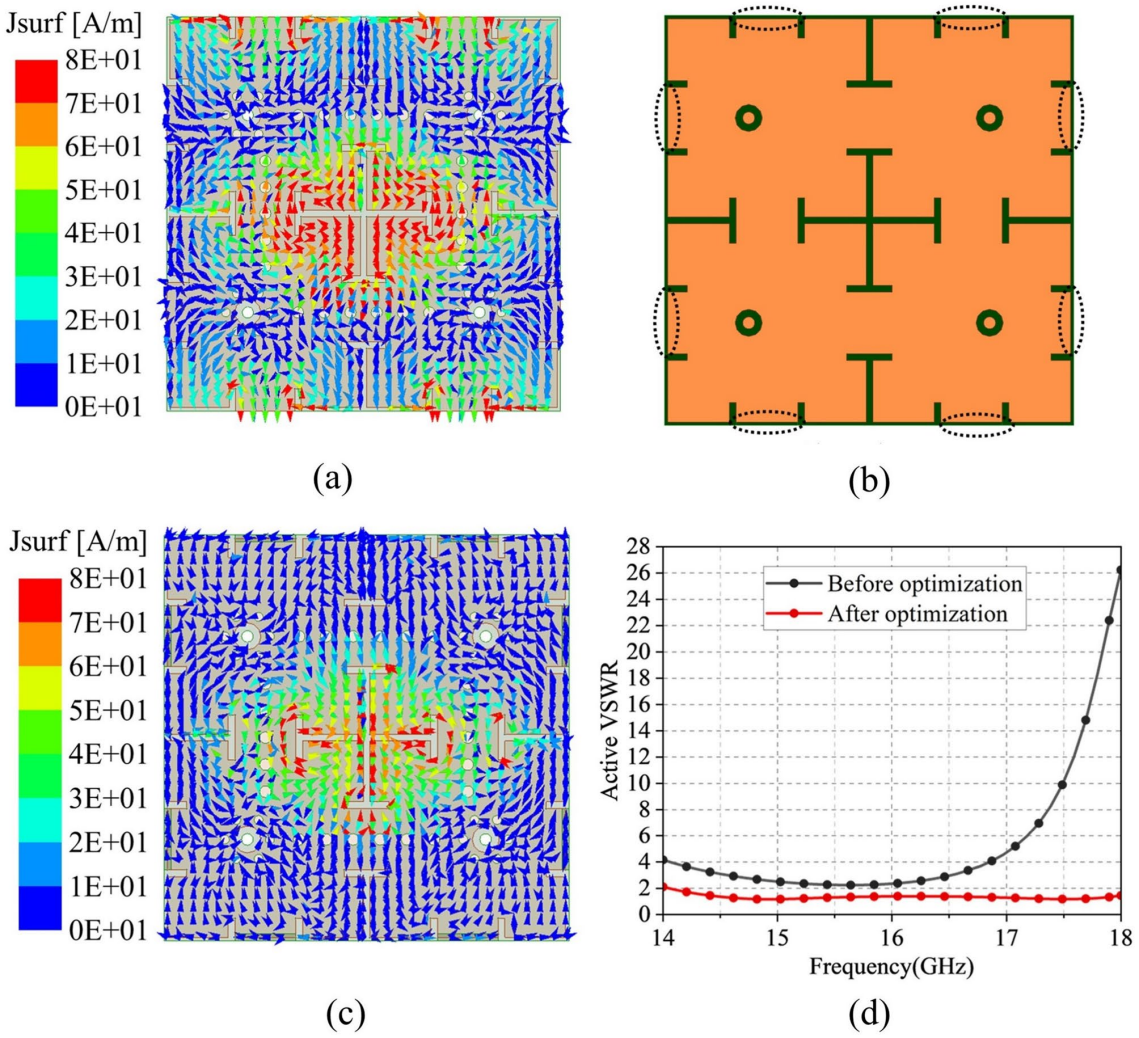
Simulation results of the slot layer in Ku-band antenna: (**a**) Surface current distribution before optimization, (**b**) Optimized slot layer structure, (**c**) Surface current after optimization, and (**d**) Comparative active VSWR performance before and after optimization.

**Fig. 6:**
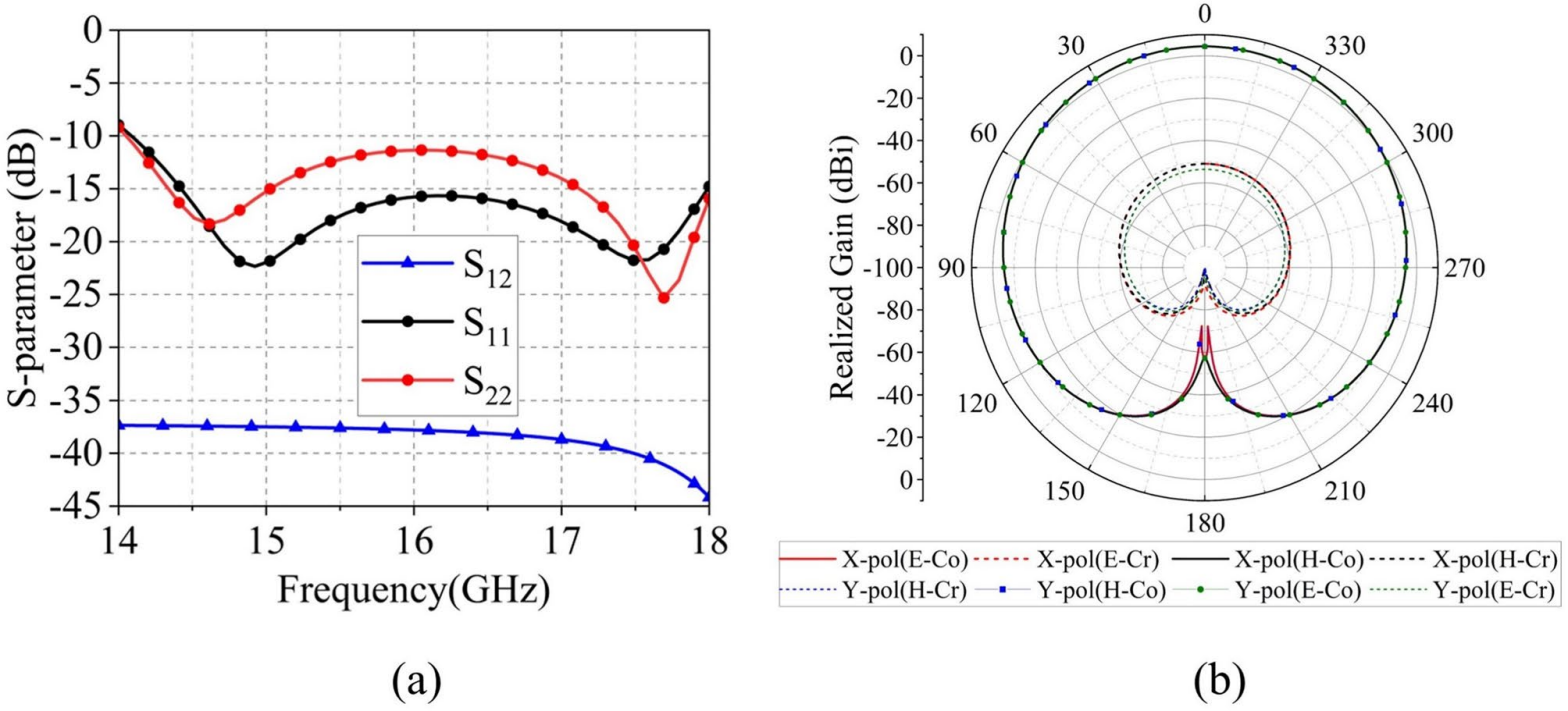
The periodic boundary simulation results of the dualpolarization Ku element: (a) S-parameter and (b) X-pol/Y-pol Realized Gain at 16GHz.

### Design of Ku-band element and structural reuse

The core design concept of the Ku-band element lies in the structural reuse of a $2\times 2$ mirrored Ka-band array. As shown in Fig. 4a, the Ka-band elements are arranged in mirrored configuration, while Fig. 4b presents the derived Ku-band element after local modifications. Specifically, cross-shaped patches are added at the centers of the second and fourth layer surfaces in the $2\times 2$ Ka-band array to improve impedance matching and enhance radiation performance. The original 0.5-mm-thick F4BM217 substrate at the bottom layer is replaced with two 0.127-mm and one 0.25-mm F4BM217 substrates to facilitate crossed feeding lines for dual-polarization. Remarkably, the DGS of the Ka-band array naturally forms the central cross-shaped feeding slot for the Ku-band element. The ADS of the Ku-band element directly inherits the $2\times 2$ Ka-band array’s ADS, achieving 100% reuse of the shared-aperture dual-band ADS.

Figure 5a displays the simulated surface current distribution on the slot layer of the model in Fig. 4b. The current distribution reveals strong edge currents at the upper and lower boundaries of the Ku-band element, similar to the Ka-band design, which would degrade the active VSWR. Consequently, the slot layer was optimized by interconnecting two originally isolated L-shaped short slots via a transverse connecting bridge, thereby forming a continuous-channel slot structure. This design modification transforms the structure into a continuous-channel slot configuration.Post-optimization simulations in Fig. 5c demonstrate significantly weakened edge currents compared with Fig. 5a, indicating reduced inter-element coupling. As evidenced in Fig. 5d, the optimized design achieves active VSWR below 2 across 14–18 GHz, showing substantial improvement over the initial configuration.

**Fig. 7:**
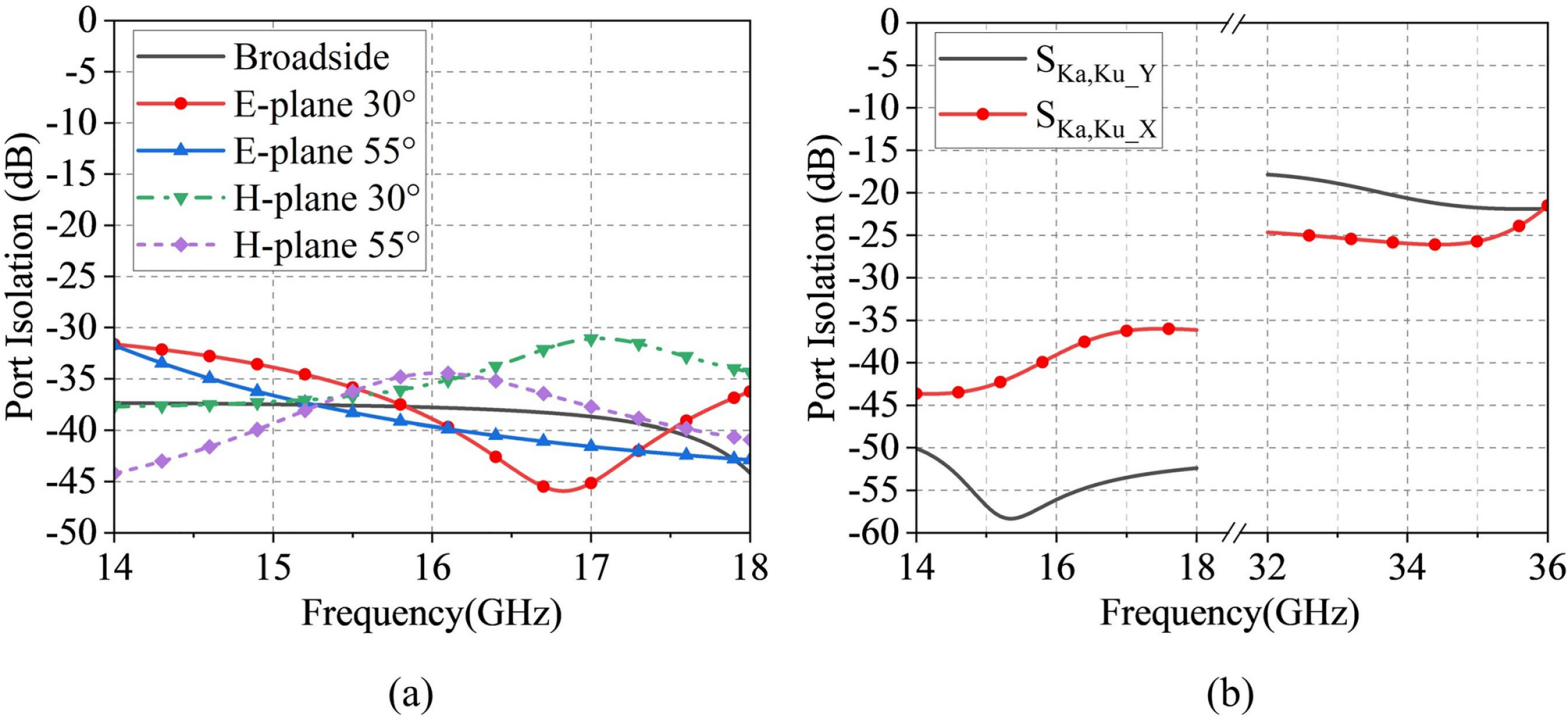
Periodic boundary SAPAA element isolation simulation results: (**a**) Port isolation between X- and Y-polarizations in Ku-band. and (**b**) Inter-band channel isolation between Ku- and Ka-band.

**Fig. 8:**
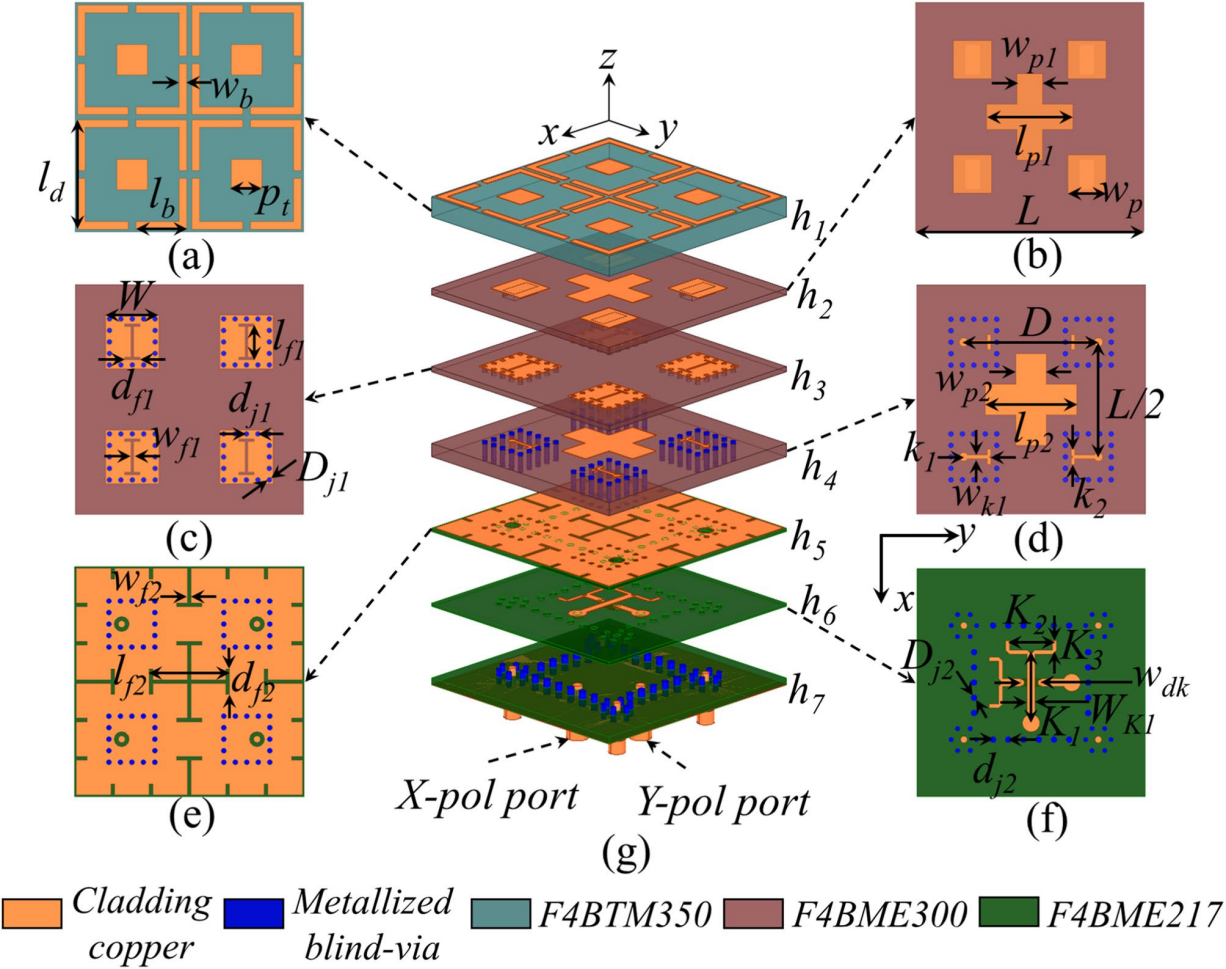
3-D exploded view and detail view of SAPAA element. Key parameters: $h_1 = 0.762$, $h_2 = 0.25$, $h_3 = 0.2$, $h_4 = 0.5$, $h_5 = 0.127$, $h_6 = 0.127$, $h_7 = 0.25$, $l_d = 4.2$, $l_b = 2$, $w_b = 0.3$, $p_t = 1.2$, $w_{p1} = 1$, $l_{p1} = 3.4$, $w_p = 1.5$, $L = 9$, $W = 2.2$, $d_{f1} = 0.6$, $l_{f1} = 1.4$, $w_{f1} = 0.1$, $d_{j1} = 0.45$, $D_{j1} = 0.2$, $D = 5.3$, $w_{p2} = 1.2$, $l_{p2} = 3.6$, $k_1 = 1$, $k_2 = 0.3$, $w_{k1} = 0.15$, $w_{f2} = 0.15$, $l_{f2} = 3$, $d_{f2} = 1$, $K_1 = 2.8$, $K_2 = 1.85$, $K_3 = 0.45$, $W_{K1} = 0.3$, $w_{dk} = 0.4$, $D_{j2} = 0.3$, and $d_{j2} = 0.6.$.

Under periodic boundary conditions, Fig. 6a shows that the dual-polarized Ku-band element maintains $\text {S}_{11}$/$\text {S}_{22}$ below $-10$ dB from 14–18 GHz with port isolation exceeding 37 dB. The radiation patterns at 16 GHz in Fig. 6b exhibit 4.4 dBi gain for both X- and Y-polarizations, with cross-polarization levels consistently below $-50$ dB. Figure 7a further demonstrates that the out-of-band port isolation between the Ka-band and Ku-band units is below $-36.3$ dB in the 14–18 GHz band and below $-17.7$ dB in the 32–36 GHz band.

**Fig. 9:**
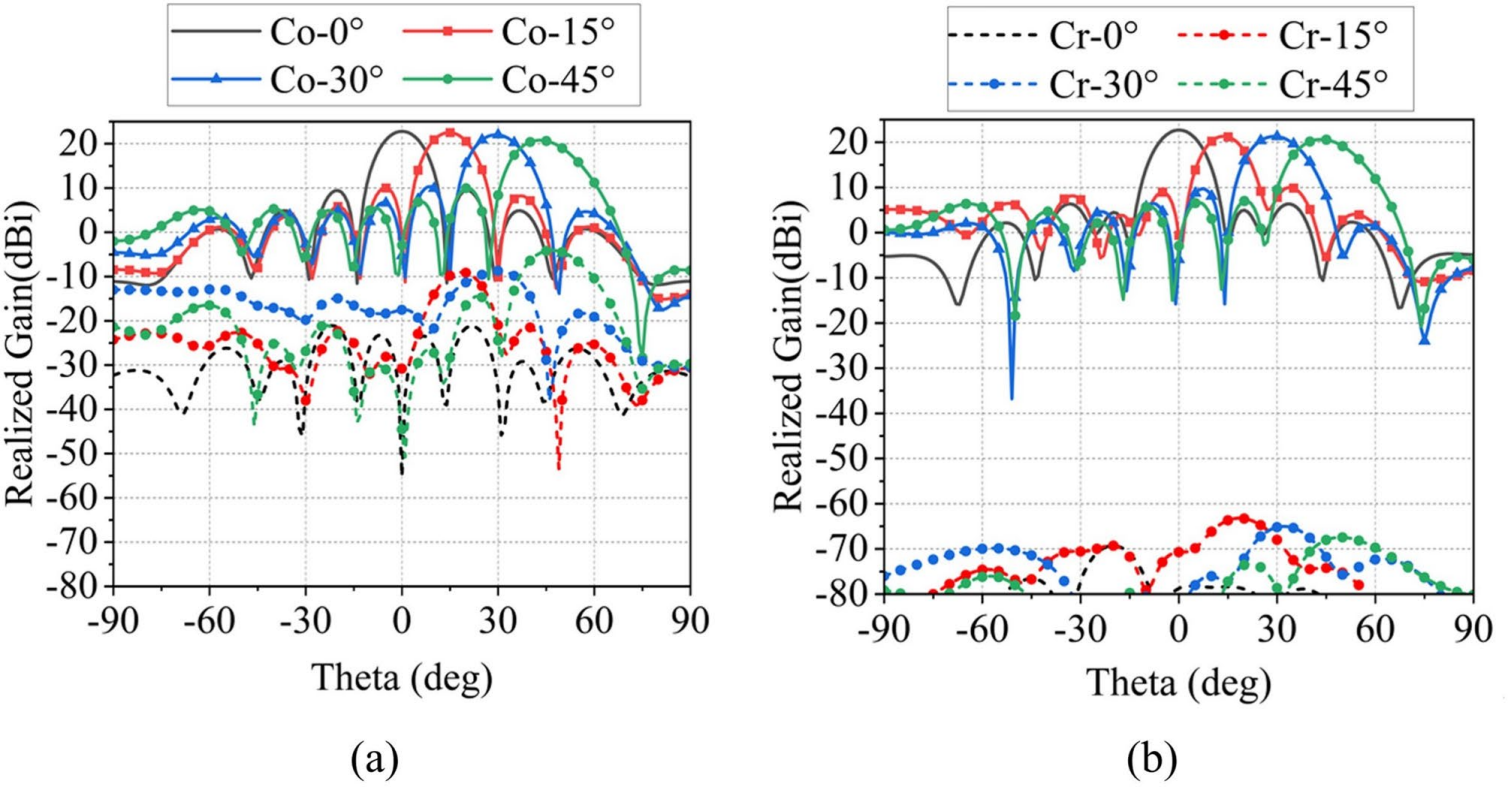
The scanning pattern of the 8 $\times$ 8 array at 34 GHz in (**a**) E-plane and (**b**) H-plane.

**Fig. 10:**
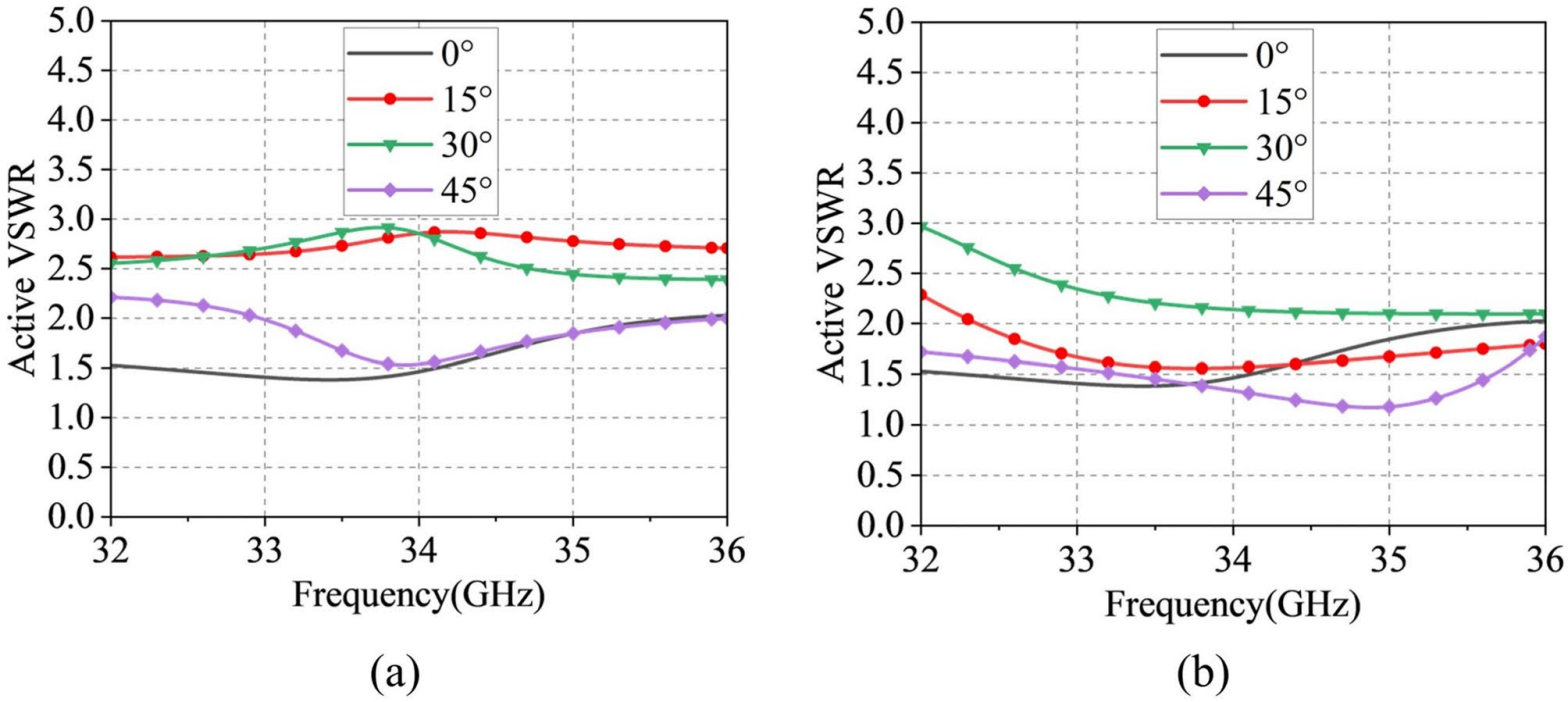
The active VSWR of the 8 $\times$ 8 array at 34 GHz in (**a**) E-plane and (**b**) H-plane.

**Fig. 11:**
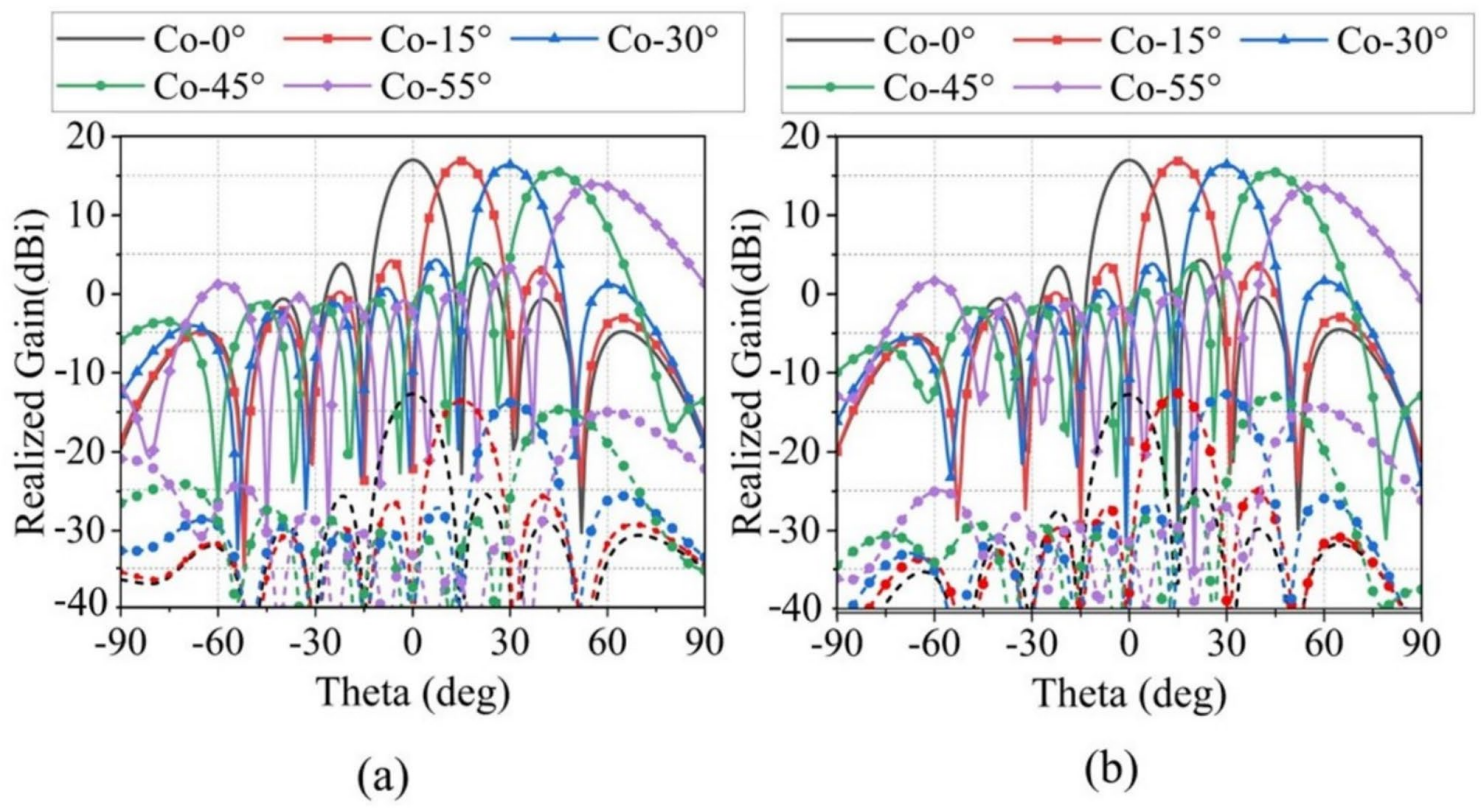
The X-pol scanning pattern of the 4 $\times$ 4 array at 16 GHz in (**a**) E-plane and (**b**) H-plane.

**Fig. 12:**
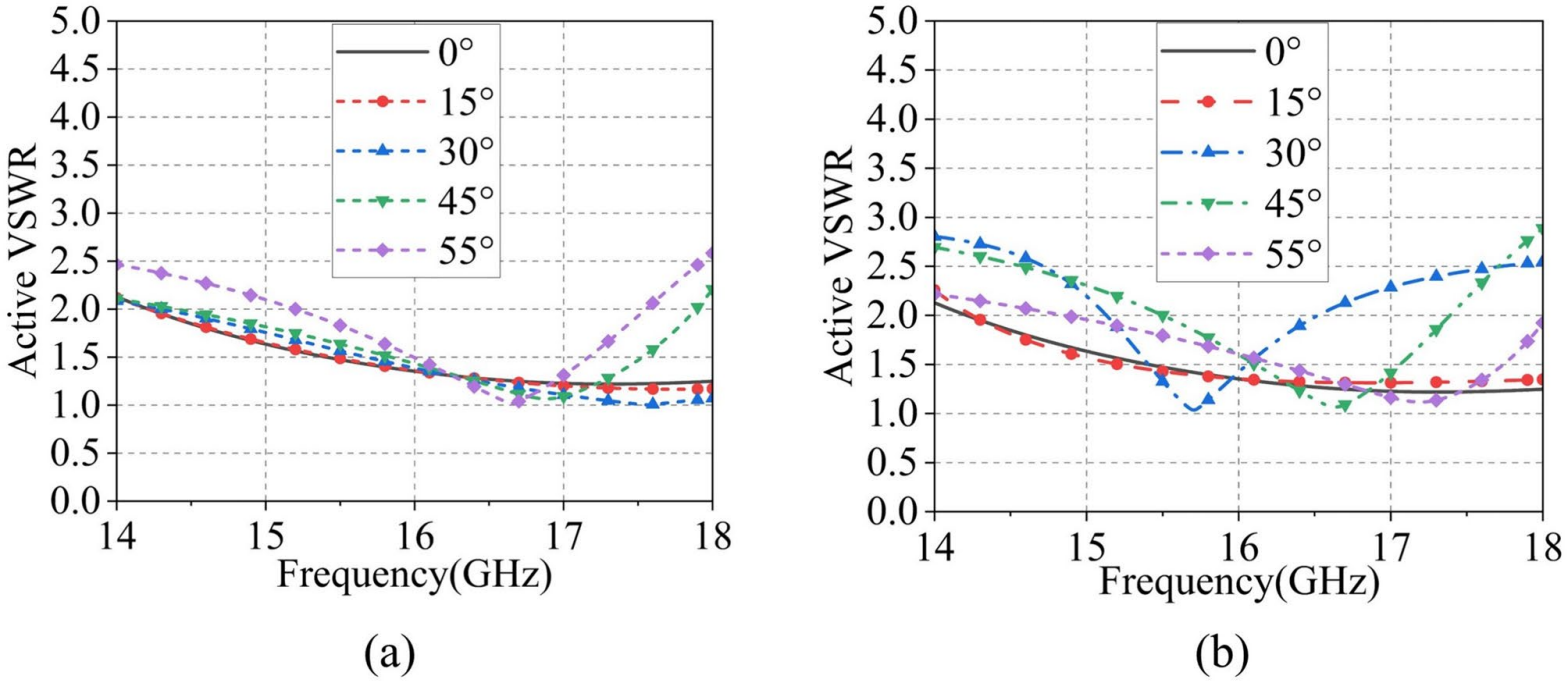
The X-pol active VSWR of the 4 $\times$ 4 array at 16 GHz in (**a**) E-plane and (**b**) H-plane.

### Design of SAPAA element

Figure 8 presents the final unit cell configuration of the proposed Ku/Ka-band SAPAA, comprising one dual-polarized Ku-band radiating element and four mirror-arranged single-polarized Ka-band radiating elements. The Ka-band patch elements are positioned at the four corners surrounding the dual-polarized Ku-band element. The entire structure utilizes a seven-layer stacked substrate configuration. The element spacing of the SAPAA is designed as 1.08$\lambda _h$, while the inter-element spacings for the Ku/Ka-band units are selected as 0.487$\lambda _1$ and 0.480$\lambda _2$, respectively ($\lambda _h$ = 36 GHz, $\lambda _1$ = 34 GHz, $\lambda _2$ = 16 GHz). This specific spacing configuration effectively suppresses grating lobes during wide-angle scanning while minimizing undesirable mutual coupling.

As illustrated in Fig. 8a–d, the main structure of single-polarized Ka-band elements occupies layers 1–4: The ADS resides on layer 1 using F4BTMS350 substrate ($\epsilon _r=3.50$, $\tan \delta =0.0024$), while the radiating patch layer, H-shaped slot layer, and feeding layer are sequentially arranged on layers 2–4 using F4BME300 substrate ($\epsilon _r=3.00$, $\tan \delta =0.0025$). The T-shaped feedlines are encapsulated within square substrate-integrated waveguide (SIW) cavities formed by metallized blind vias. Layers 5–7 utilize F4BME217 substrate ($\epsilon _r=2.17$, $\tan \delta =0.0014$), with Fig. 8e showing the DGS for Ka-band elements on layer 5, effectively suppressing inter-element coupling.

The dual-polarized Ku-band element’s main structure is shown in Fig. 8b,d–f. The Ku-band radiating patches occupy layers 2 and 4, while the Ka-band DGS on layer 5 is repurposed as a cross-shaped feeding slot for Ku-band operation. Figure 8f,g reveal that two T-shaped feedlines are primarily distributed on layer 6’s top surface, where a segment of the Y-polarized feedline detours through metallized vias from lower layers to avoid the X-polarized feedline. Similar to the Ka-band feeding structure, the Ku-band dual-polarized feeding network is also encapsulated within square SIW cavities.

## Simulation of SAPAA

To validate the designed shared-aperture phased array antenna, an $4 \times 4$ Ku-band phased array and an $8 \times 8$ Ka-band phased array were modeled and simulated in CST Microwave Studio. The simulated results of the Ka-band array are presented in Fig. 9. Figure 9a,b illustrate the E-plane and H-plane far-field radiation patterns at 34 GHz for different scan angles, respectively. The array achieves a peak gain of 22.7 dBi at 34 GHz, with a gain reduction of less than 2 dB at a $45^\circ$ scan angle. Under the extreme $45^\circ$ scan condition, the sidelobe levels (SLLs) are below $-10$ dB and $-13$ dB for the E-plane and H-plane, respectively, while the cross-polarization levels are better than $-25$ dB and $-80$ dB, respectively. These results demonstrate that the array maintains acceptable sidelobe performance and excellent cross-polarization discrimination over a wide scanning range. Figure 10a,b demonstrate that the active VSWR of Ka-band elements remains below 3 for both E- and H-plane scanning up to $\pm 45^\circ$.

Owing to the structural symmetry of the SAPAA unit, the scanning performance under Y-polarization port excitation is nearly identical to that under X-polarization. Therefore, only the scanning characteristics of the $4\times 4$ Ku-band array with X-polarization feeding are presented in Fig. 11. Figure 11a,b show the far-field patterns at 16 GHz for scan angles from $0^\circ$ to $55^\circ$, achieving a peak gain of 17.13 dBi. Benefiting from the high symmetry of the dual-polarized feeding structure, the scanning performance exhibits high consistency between the E- and H-planes. In both principal planes, the maximum gain degradation is limited to 1.55 dB and 3.3 dB at $45^\circ$ and $55^\circ$ scan angles, respectively. At the extreme $55^\circ$ scan angle, the SLLs are below $-11$ dB in both E- and H-planes, while the cross-polarization levels are better than $-29$ dB and $-28$ dB, respectively. The array maintains acceptable sidelobe characteristics and good polarization purity even under large-angle scanning conditions. As evidenced in Fig. 12a,b, the active VSWR of Ku-band elements stays below 3 throughout $\pm 55^\circ$ scanning in both principal planes.

**Fig. 13:**
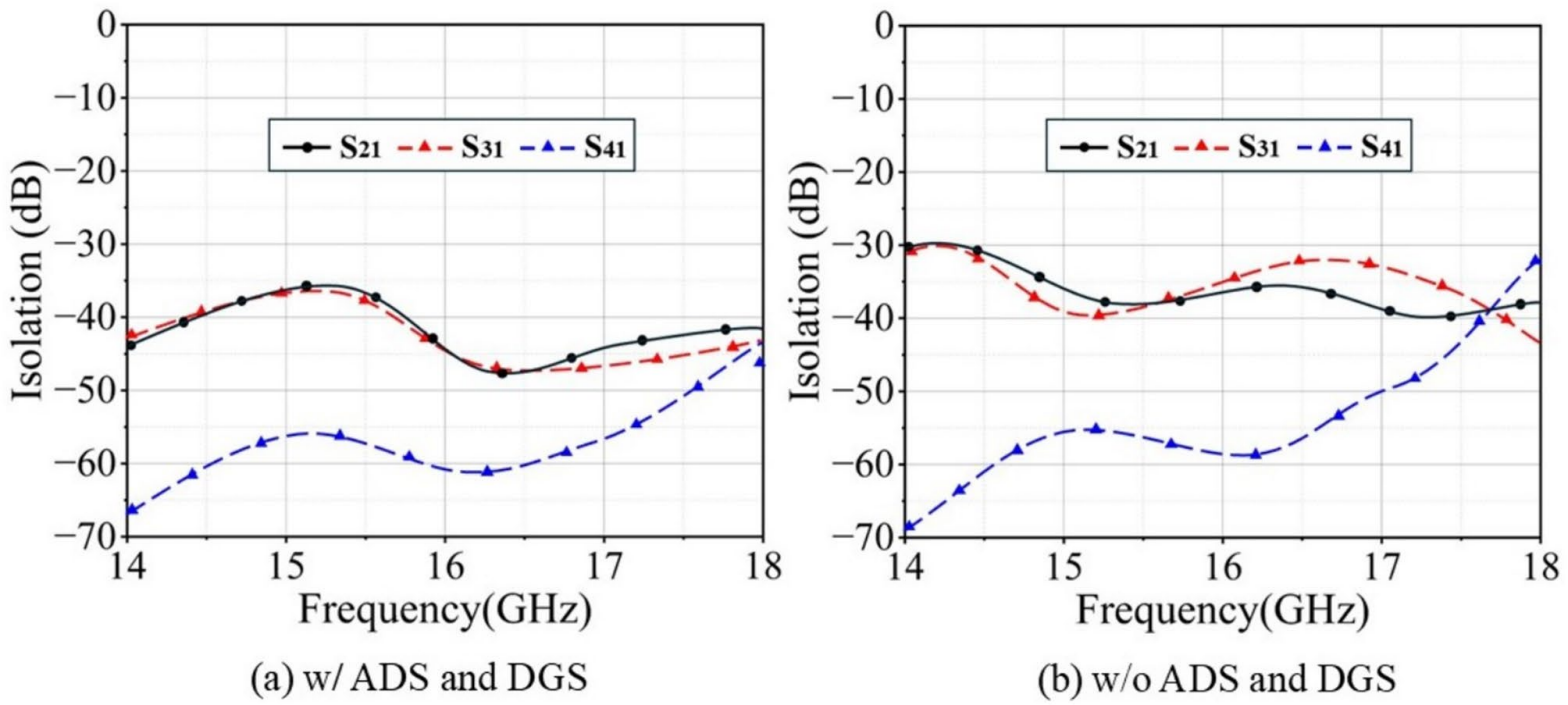
Simulated isolation enhancement comparison of 2 $\times$ 2 Ku-band unit groups within the SAPAA.

**Fig. 14:**
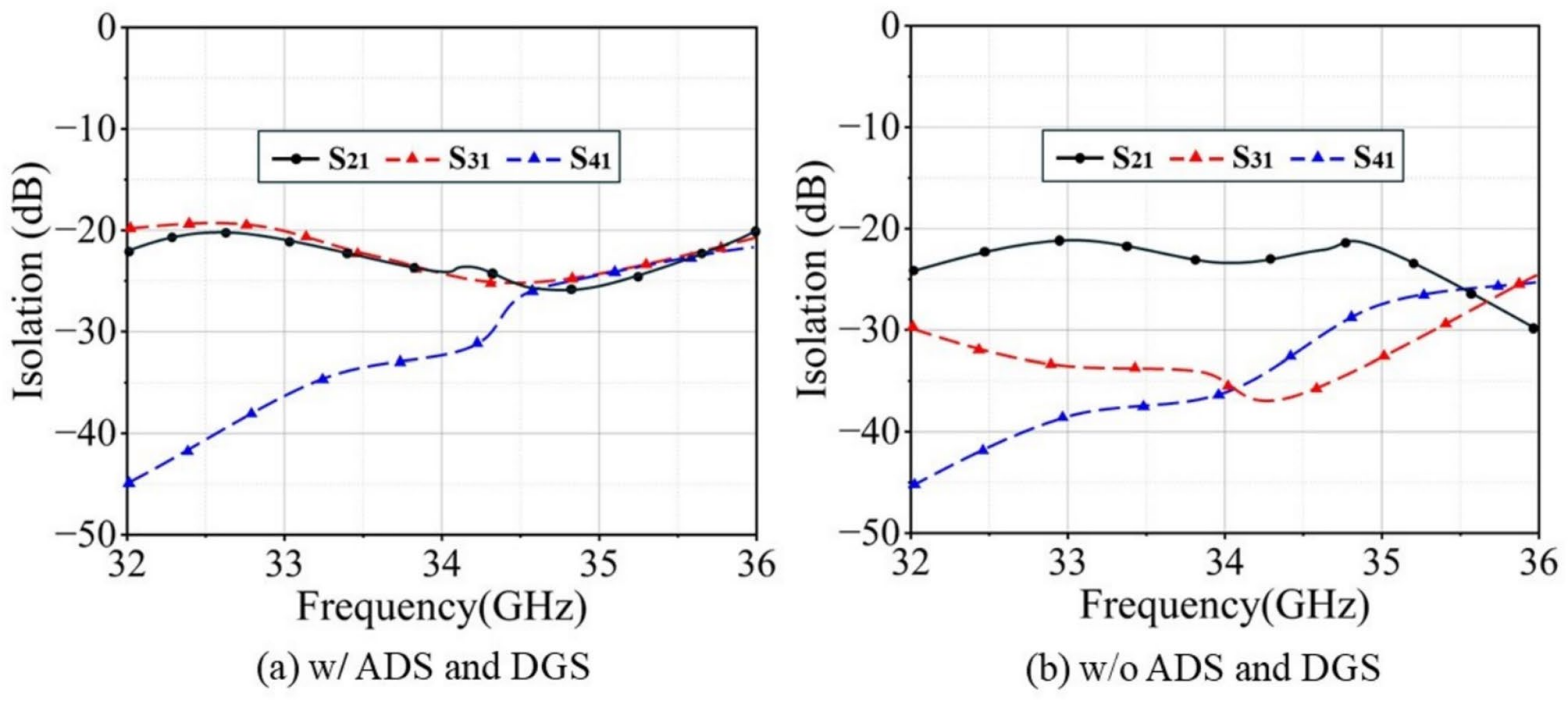
Simulated isolation enhancement comparison of 2 $\times$ 2 Ka-band unit groups within the SAPAA.

Figures 13 and 14 present the simulated isolation curves of adjacent $2 \times 2$ Ku-band and Ka-band unit groups within the SAPAA, respectively, along with a comparison to the isolation performance after removing the hybrid decoupling structure. The results indicate that the in-band isolation exceeds 18 dB for the Ku-band and 35 dB for the Ka-band under the co-aperture configuration. A comparison between Fig. 13a,b, as well as between Fig. 14a,b, clearly demonstrates that the proposed hybrid decoupling structure significantly enhances the in-band isolation performance of the antenna in the respective frequency bands.

## Antenna array experiment

**Fig. 15:**
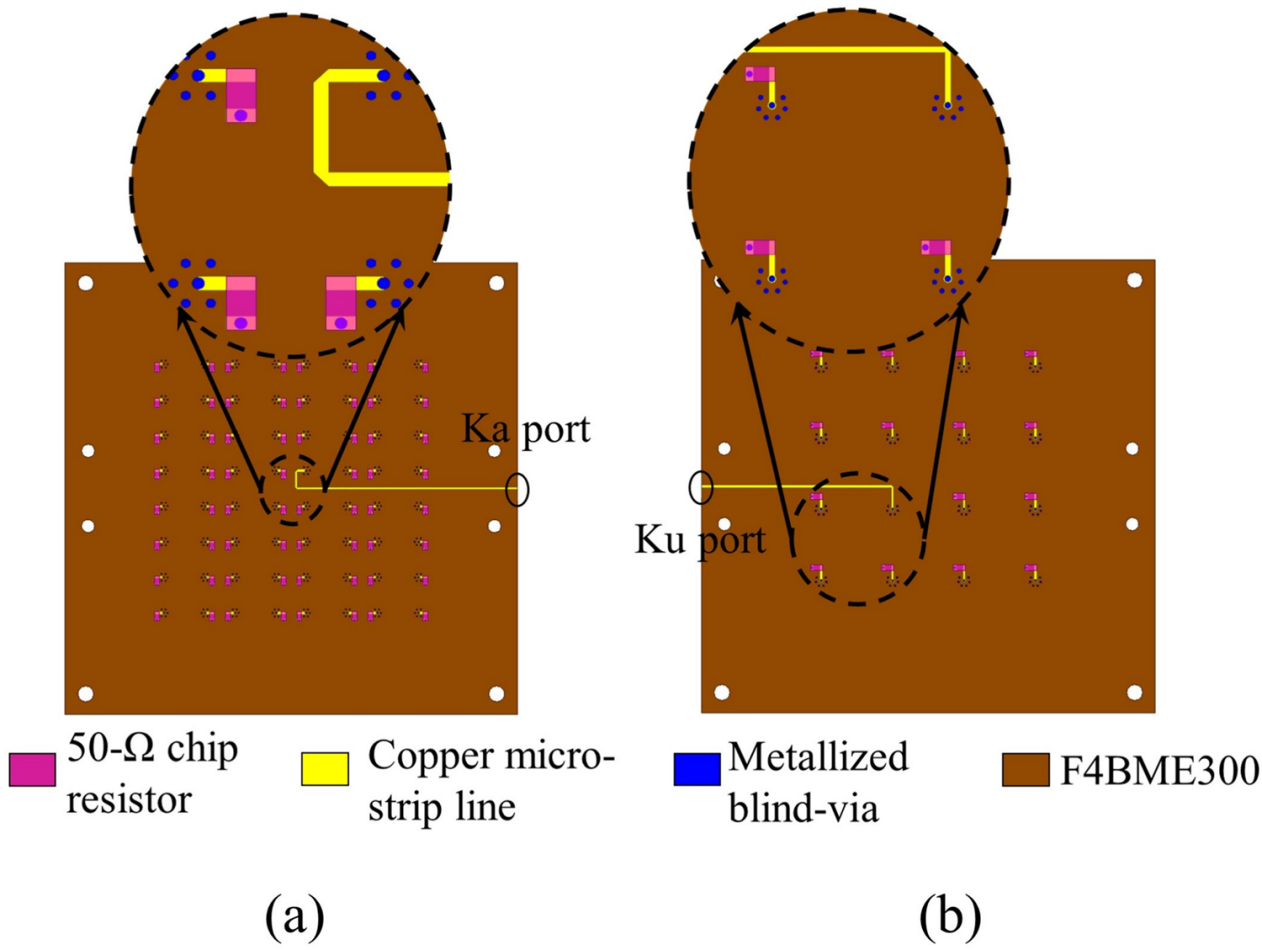
Bottom-view layout of the finalized (**a**) 8 $\times$ 8 Ka-band array design and (**b**) 4 $\times$ 4 Ku-band array design ready for fabrication.

**Fig. 16:**
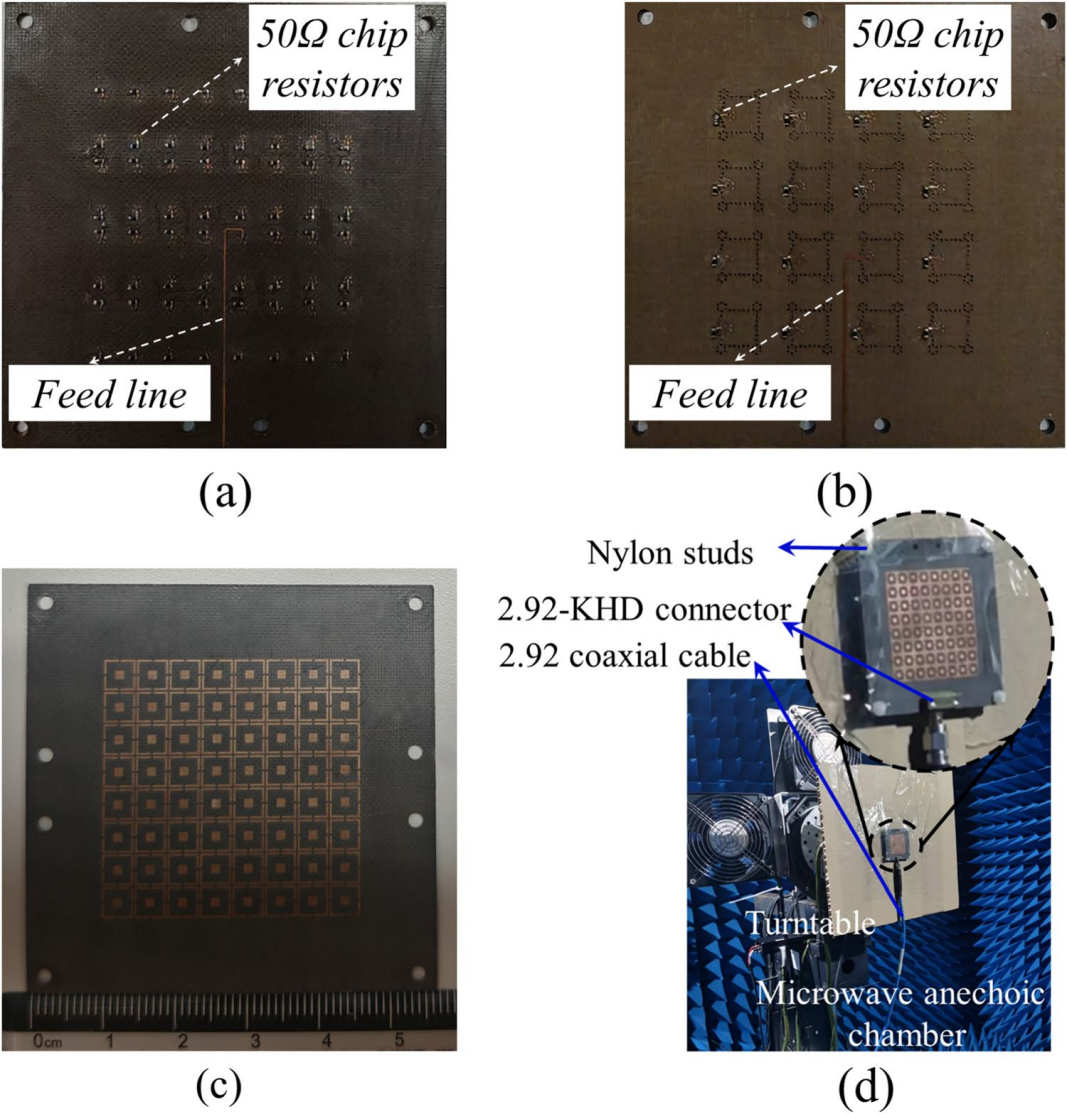
(**a**) Bottom view of the 8 $\times$ 8 Ka-band array, (**b**) Bottom view of the 4 $\times$ 4 Ku-band array, (**c**) Top view of the SAPAA and (**d**) Anechoic chamber measurement.

To address the challenges of high integration difficulty and cost between miniature microwave connectors and planar millimeter-wave array feeding networks, a compromise solution following References^3,9^ is adopted, implementing the following specific fabrication approach for the SAPAA: The fabricated antenna consists of 11 substrates, separately designed for testing Ka/Ku-band phased arrays. This includes shared layers 1–3, plus two distinct feeding layer sets (layers 4–7) specifically for Ku-band and Ka-band arrays (only differing in the bottommost layer). Figure 15 illustrates the bottom-side proposed structure of the 8 $\times$ 8 Ka-band array and 4 $\times$ 4 Ku-band array, showing their feeding conditions where only the center element is actively fed while other elements are terminated with 50$\Omega$ resistors for impedance matching. The bottom-view photographs of the physical prototypes are presented in Fig.  16a,b. Notably, since the 4 $\times$ 4 Ku-band array shares identical dimensions with the 8 $\times$ 8 Ka-band array, their top views are identical as demonstrated by the common top-view photograph in Fig. 16c.

**Fig. 17:**
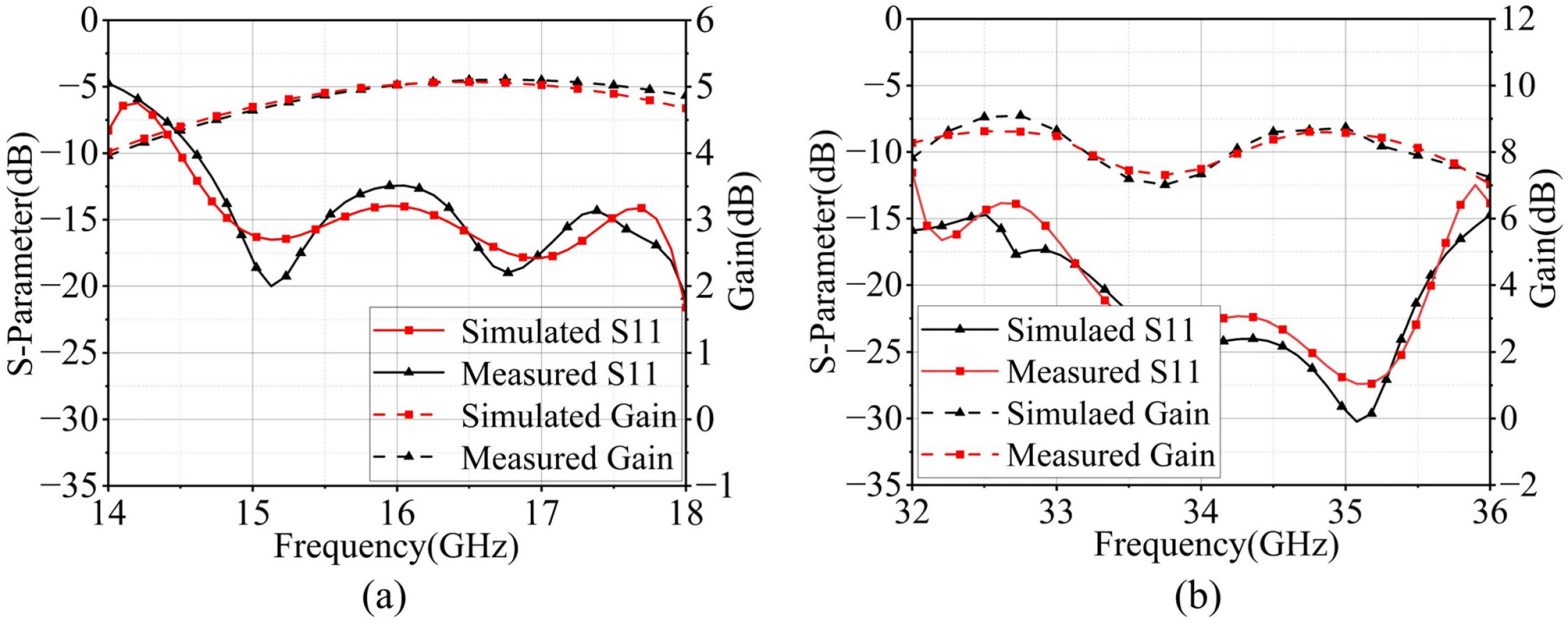
The simulated and measured reflection coefficients of the (**a**) 8 $\times$ 8 Ka-band array and (**b**) 4 $\times$ 4 Ku-band array.

Figure 16d displays the fabricated array prototype and anechoic chamber test setup: The prototype is mounted on a positioning turntable and fed through a 2.92-KHD connector with 2.92 coaxial cables. The simulated and measured reflection coefficients and gains for the center elements of both $8 \times 8$ Ka-band and $4 \times 4$ Ku-band arrays are compared in Fig. 17a,b. Although discrepancies exist between the measured and simulated results, they nevertheless remain within acceptable limits when accounting for the impedance mismatch introduced by the 2.92-KHD connector, as well as the dielectric constant tolerances and stacking assembly tolerances of the multi-layer substrate.

It is noteworthy that although this work is based on an $8\times 8$ (Ka-band)/$4\times 4$ (Ku-band) array prototype, the proposed methodology remains applicable for large-scale array expansion. The array elements are arranged in a strictly periodic layout, and key performance metrics such as isolation and scanning gain have been verified through simulations employing periodic boundary conditions under the infinite array assumption. This modeling approach inherently captures the electromagnetic behavior of large-scale arrays. Furthermore, the adopted hybrid decoupling structure operates within individual unit cells, meaning its coupling suppression mechanism is independent of the array scale. Structural reuse technology ensures that decoupling between Ku/Ka-band units is achieved through localized inter-layer isolation (Fig. 8d,g), a design feature independent of the overall array dimensions. For practical implementation, the feeding network employs a layered packaging architecture, where Ka-band and Ku-band feeding lines are isolated within separate SIW cavities (Fig. 8d,g), effectively mitigating routing crosstalk in large-scale arrays.

Table 1 presents a comprehensive comparison between the proposed SAPAA and existing designs operating in similar frequency bands. For fair evaluation, all scanning angles were assessed under identical simulation conditions. The innovative planar stacked-patch configuration combined with structural multiplexing technology enables an ultra-low profile of 0.28$\lambda _h$, achieving a 48–65% height reduction compared to designs in ^25,26,28^. The ADS- and DGS-based hybrid-decoupling mechanism significantly enhances beam-scanning performance, demonstrating $\pm 55^\circ$ and $\pm 45^\circ$ scanning ranges in Ku-band and Ka-band respectively—a notable improvement over the results reported in ^9^. Furthermore, the proposed design maintains superior channel isolation exceeding 35 dB in Ku-band and 18 dB in Ka-band. These combined advantages in scanning capability, structural compactness, and isolation performance establish our SAPAA as a highly competitive solution for multiband integrated communication systems.

**Table 1:** Comparison between the proposed SAPAA and other works.

Reference	Frequency (GHz)	Profile	Polarization	Isolation (dB)	Gain reduction (dB)	Scan range
^25^	16	$4.70\lambda _h$	Single	28	<1.8	$\pm 40^\circ$
	34.7		Single	70	<1.8	$\pm 40^\circ$
^26^	16	$1.73\lambda _h$	Single	40	<3.3	$\pm 50^\circ$
	35		Single	18	<3.4	$\pm 40^\circ$
^28^	16	$>1.14\lambda _h$	Dual	27	<3.1	$\pm 50^\circ$
	34.5		Single	15	<2.2	$\pm 50^\circ$
^9^	15.5	$0.39\lambda _h$	Dual	27	<2.3	$\pm 45^\circ$
	34.5		Single	13	<1.5	$\pm 30^\circ$
This Work	16	$0.28\lambda _h$	Dual	35	<3.3	$\pm 55^\circ$
	34		Single	18	<1.55	$\pm 45^\circ$

## Conclusions

This paper proposes a low-profile, wide-angle-scanning Ku/Ka dual-band shared-aperture phased array antenna based on hybrid-decoupling techniques. The design employs mirror-arranged Ka-band single-polarized elements as the foundational stacked architecture, while innovatively integrating the Ku-band element’s dual-layer radiating patches and feeding structure through structural reuse technology. Co-optimization of decoupling structures and feeding networks effectively suppresses inter-band coupling, achieving an ultra-thin profile of $0.28\lambda _H$, wide scanning ranges of $\pm 55^\circ$ (Ku-band) and $\pm 45^\circ$ (Ka-band), while maintaining high isolation of 35 dB (Ku-band) and 18 dB (Ka-band). A prototype antenna was fabricated to validate the simulations, with measured results demonstrating the design’s effectiveness. This work provides an innovative solution combining high performance and compactness for drone swarm and other dynamic communication systems.

## Data Availability

The datasets used and/or analyzed during the current study are available from the corresponding author upon reasonable request.
